# Chromosomal abnormalities in 163 Tunisian couples with recurrent miscarriages

**DOI:** 10.11604/pamj.2017.28.99.11879

**Published:** 2017-09-29

**Authors:** Wiem Ayed, Islem Messaoudi, Zouhour Belghith, Wajih Hammami, Imen Chemkhi, Nabila Abidli, Helmy Guermani, Rim Obay, Ahlem Amouri

**Affiliations:** 1 Department of Histology and Cytogenetics, Institut Pasteur de Tunis, Tunisie; 2 Faculty of Medecine of Tunis, University of Tunis El Manar, Tunisia; 3 Laboratory of Biomedical Genomics and Onco-genetics LR11IPT05, Institut Pasteur de Tunis, Tunisia

**Keywords:** Recurrent miscarriage, chromosomal abnormalities, karyotype

## Abstract

Recurrent miscarriage (RM) is defined as three or more consecutive pregnancy losses before 24 weeks of gestation. Parental chromosomal abnormalities represent an important etiology of RM. The aim of the present study was to identify the distribution of chromosome abnormalities among Tunisian couples with RM referred to the Department of Cytogenetic at the Pasteur Institute of Tunis (Tunisia) during the last five years. Standard cytogenetic analysis was carried out in a total of 163 couples presenting with two or more spontaneous abortions. Karyotypes were analyzed by R-banding. We identified 14 chromosomal abnormalities including autosomal reciprocal translocation, Robertsonian translocation, inversion, mosaic aneuploidy and heteromorphysm. The overall prevalence of chromosomal abnormalities was 8.5% in our cohort. This finding underlies the importance of cytogenetic investigations in the routine management of RM.

## Introduction

Recurrent miscarriages are post implantation failures in natural conception. They are also termed as habitual abortions or recurrent pregnancy losses [1]. Recurrent miscarriage (RM) is defined by some authors as three or more pregnancy loss before 20-24 weeks and was considered as a distinct disease entity [2-7]. In 2005, the European Society of Human Reproduction and Embryology (ESHRE) introduced a revised terminology regarding early pregnancy events. A pregnancy loss that occurs after a positive urinary human chorionic gonadotropin (hCG) or a raised serum β-hCG but before ultrasound or histological verification is defined as a “biochemical loss”. In general, these occur before 6 weeks of gestation. The term clinical miscarriage is used when ultrasound examination or histological evidence has confirmed that an intrauterine pregnancy has existed. Clinical miscarriages may be subdivided into early clinical pregnancy losses (before gestational week 12) and late clinical pregnancy losses (gestational weeks 12 to 21). There is no consensus on the number of pregnancy losses needed to fulfill the criteria for recurrent miscarriage (RM), but ESHRE guidelines define RM as three or more consecutive pregnancy losses before 22 weeks of gestation [1].

Recurrent miscarriage occurs in approximately 3% of women with diagnosed pregnancies [8] and affects about 1-3% of women during their reproductive years [9]. Various etiologies, either alone or in combination, have been proposed to contribute to pregnancy loss including, uterine malformations, infections, maternal thrombophilic disorder, immune dysfunction, various endocrine disturbances and parental chromosomal anomalies [10]. Among the various etiologies, genetic factors appear to be highly associated with reproductive loss [11, 12]. In 50% of the couples, no specific cause can be identified and in this situation, they are regarded as having idiopathic or unexplained RM [12]. Between 29% and 60% of cases, RM could be caused by chromosomal aberrations in the embryo [13].

It is generally assumed that chromosomal anomalies found in the fetus were due to a balanced aberration in one of the parents being inherited by the offspring in an unbalanced form [13]. A chromosomal abnormality in one partner is found in 3% to 6% of RM couples, which is ten times higher than the background population [7]. Changes in the karyotypes include balanced reciprocal translocation, robertsonian translocation, gonosomal mosaic and inversions [9]. The aim of this study was to identify the types of chromosomal abnormalities in couples with two or more recurrent miscarriage referred to Cytogenetic Department of Pasteur Institute of Tunis.

## Methods

Between January 2011 and December 2016, 163 couples with ≥ 2 recurrent miscarriage were referred to Cytogenetic Department of Pasteur Institute of Tunis, from different parts of the country. All of these patients underwent a complete clinical assessment, including complete medical and gynaecological history in order to exclude immunologic effects, uterine malformations and other causes of recurrent abortions. Written informed consent was obtained from all participants.

Metaphase chromosome preparations from the peripheral blood cultures were made according to standard cytogenetic protocols. Cytogenetic analysis was performed by RHG banding. Twenty metaphases were analyzed in all the patients but in cases of abnormalities and mosaicism, the study was extended to 50 metaphases. Chromosomal abnormalities were reported according to the International System for Human Cytogenetic Nomenclature (ISCN 2009).

X^2^ and *FISHER tests* and linear regression study were used to examine the significance of association (*P*<0.05) and performed with Epi Info 7.

## Results

**Figure 1:**
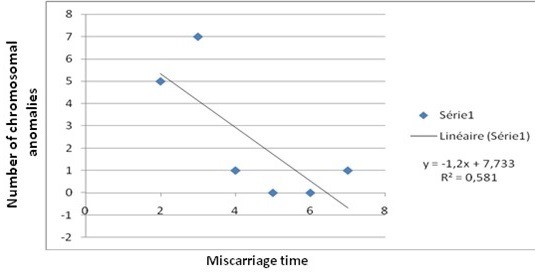
The relation between chromosomal abnormalities and miscarriage times

A total of 163 couples (326 cases) with history of recurrent miscarriage were examined. The median age of female partner was 31.94 years ± 0.70 where as the median age of male partner was 36.61± 4.94. The number of recurrent abortion varied from 2 to 7 abortions/couple. Chromosomal abnormalities were present in 14 cases (8,5%). It was found in 6.7% (5/74) of the couples with a history of two abortions, in 10.7% (7/65) with three abortions and in 8.3% (2/24) with four or more abortions. Women were more frequently affected than men, with a prevalence of 7% and 0.6 % respectively (P<0.05). No couple presented an abnormal karyotype in both partners. The number of chromosomal abnormalities decreases significantly with the times of miscarriage (R2 = 0,58 ; P=0 03 ; DF=5) (Figure 1).

Among 14 abnormalities, 6 cases showed structural aberrations and 4 cases presented numerical anomalies. Four other cases presented heteromorphism which included qh+ (secondary constriction increase) in chromosome 9, and s+ (satellite increase) in chromosomes 13, 14 and 22 (Table 1). The majority of the structural abnormalities were balanced reciprocal translocations whereas, robertsonian translocation was found in only one patient involving chromosomes 13 and 14 (Table 1).

**Table 1:** List of chromosomal abnormalities identified in 163 couples with recurrent miscarriage (RM)

Anomalies	Karyotype	Numbre	Sex	Age	Nb of RM
Reciprocal translocation	46,XX,t(4;10)(q28;q25)	1	F	31	3
	46,XX,t(14;18)(q21:p11)	1	F	NI	3
	46,XX,t(14;18)(q13:p22)	1	F	NI	7
Robertsonian translocation	45,XX,rob (13;14)(q10;q10)	1	F	30	3
Inversion	46,XX,inv (11)(p14q13)	1	F	22	3
	46,XX,inv (12)(p12q12)	1	F	26	2
Aneuploidy	46,XX[17]/45,X[5]	1	F	40	3
	46,XX [47]/45,X[3]	1	F	30	3
	46,XX[22]/45,X[3]	1	F	33	4
	46,XX[24]/ 45,X[3]/47,XXX[1]	1	F	41	2
Heteromorphism	46,XX,9qh+	1	F	31	3
	46,XY,13s+	1	M	35	2
	46,XX,22s+	1	F	20	2
	46,XX,14s+	1	F	35	2

F: female; M: male; Nb: number; RM: recurrent miscarriage; NI: non identified

## Discussion

Recurrent miscarriage continues to be a challenging reproductive problem for the patient and clinician. More than 50% of the spontaneous miscarriages are caused by chromosomal abnormalities in the embryo or fetus [14]. The genetic etiology for multiple spontaneous pregnancy loss includes an unbalanced chromosome rearrangement, which may be the result of one parent being a carrier for balanced chromosome rearrangement [15].

Several studies have been carried out to determine the prevalence of chromosomal aberrations among couples with recurrent miscarriage. This prevalence ranges widely from 2.7% [16] to 13.9% [15]. In our study, we found that the incidence of chromosomal abnormalities among couples with two or more miscarriages was 8,5%. It was close to that reported by Frikha et al [17], higher than reported by Flynn et al [12], Dutta et al [2], Elghezal et al [3] and lower to that described by and Mozdarani et al [15]. These differences may be related to sample size and to different criteria (Table 2).

**Table 2:** Frequency of chromosomal abnormalities in our study and other populations

Studies	Numberof couples	Inclusion’s criteria	Incidence %	Populations
Our study	163	≥ 2 RM	8,5	Tunisia
Flynn et al. 2013 [3]	795	≥ 2 RM <24 WA	3,52	China
Frikha et al. 2012 [17]	168	≥ 2 RM <24 WA	8,92	Tunisia
El-Dahtory et al. 2011 [19]	73	≥ 2 RM	6,1	Egypt
Dutta et al. 2010 [2]	1162	≥ 2 RM <24 WA	3,35	India
Mozdrani et 2008 [15]	79^+^	≥ 3 RM	13,9	Iran
Elghezal et al. 2007 [3]	1400	≥ 2 RM <24 WA	6,93	Tunisia
Stephenson et al.2006 [16]	1893	≥ 2 RM <20 WA	2,7	Canada
Godding et al. 2004 [27]	1324	≥ 2 RM <20 WA	3,09	Netherland

RM: Recurrent Miscarriage; WA: Weeks of Amenorrhea; ^+^:79 patients

In our study, the number of chromosomal abnormalities decreases significantly with the time of miscarriage, like found by Frikha et al [14], contrarily to that reported in the literature which is significantly increasing [18, 19]. However, Carp and all, showed that the prevalence of chromosomal aberrations was independent of the number of previous abortions [13]. Overall, chromosomal aberrations are the cause of 50% of first trimester spontaneous abortions [20]. In Our study, all patients with chromosomal abnormalities presented spontaneous abortions in first trimester.

As has also been reported in other studies, structural chromosomal aberrations were the most common chromosomal abnormalities detected in this study (6/14). Literature reported that only 0.7% of normal population were with structural aberrations, 2.2% occurred in cases who suffered abortion once, the rate increased to 4.8% in cases with 2 times of abortions and was 5.2% in those with 3 times of abortions [21]. The most frequent structural chromosome abnormalities in recurrent miscarriage are translocations (reciprocal translocation (62%), robertsonian translocation (16%), inversions (16%) and deletions and duplications (3%) [22]. The prevalence of balanced translocation among couples with recurrent abortion in different studies ranges from 0-31%. The reason of this extent variation is not clear [18, 23].

The structural chromosome abnormalities that we encountered were divided into balanced reciprocal chromosome translocations (3/14), Robertsonian translocation (1/14) and inversions (2/14). The distribution of structural chromosomal rearrangement in our study is similar to that reported worldwide.

Among the human chromosome, chromosome no.9 appears with the high frequency of structural heteromorphism, which is a natural variation that occur 1-2% among the individuals in the general population and transmitted through family as mendelian trait [22]. In our research, the rate of heteromorphism was 2,4% and included 9qh+and s+. Whether heteromorphism can cause disease is controversial. Ueharas et al [24], reported that inv (9) was related with infertility and multiple times of abortions. Rodriquez et al [25], regarded that Yq+ did not relate with birth failure while Genest et al, considered Yq+ might cause habitual abortion [21, 26]. Further analysis in “control” Tunisian population must be achieved to conclude about the impact of these heteromorphisms.

Eventually, our study agree with several previous studies indicating an increase in the number of balanced chromosomal translocation in couple with two or more abortion compared with general population. Numerical chromosomal aberrations are less frequently encountered among couples with RM. Those aberrations are usually in form of sex chromosomal aneuploidy, and they occur in a low frequency (0.15% of cases) [27]. In our study, we found 4/14 cases with X chromosome mosaicism. Monosomy X cell’s rate varied between 6% and 22%.

## Conclusion

Our study confirm that chromosomal abnormality is one of the important factors contributing to RM. Chromosomal analysis is a necessary part of the etiological research in couple with recurrent miscarriage. The identification of chromosomal abnormalities facilitated the counseling and the appropriate management.

### What is known about this topic

- A chromosomal abnormality in one partner is found in 3% to 6% of RM couples, which is ten times higher than the background population. The incidence of chromosomal abnormalities among couple with RM ranges widely from 2.7% to 13.9%;
- The number of chromosomal abnormalities is significantly increasing with the time of miscarriage;
- Structural chromosomal aberrations were the most common chromosomal abnormalities.

### What this study adds

- In our study, we found that the incidence of chromosomal abnormalities among couples with two or more miscarriages was 8,5%;
- In our study, the number of chromosomal abnormalities decreases significantly with the time of miscarriage;
- Among all abnormalities, structural aberrations were the most but 4 patients presented numerical anomalies and four other cases presented minor anomalies heteromorphism which included qh+ (secondary constriction increase) in chromosome 9, and s+ (satellite increase) in chromosomes 13, 14 and 22.

## Competing interests

The authors declare no competing interests.
